# Advances in Multivariate and Multiscale Physiological Signal Analysis

**DOI:** 10.3390/bioengineering9120814

**Published:** 2022-12-16

**Authors:** Antonio Lanata, Mimma Nardelli

**Affiliations:** 1 Department of Information Engineering, University of Florence, Via Santa Marta 3, 50139 Florence, Italy; 2 Bioengineering and Robotics Research Centre “E. Piaggio” and Dipartimento di Ingegneria dell’Informazione, University of Pisa, Largo Lucio Lazzarino 1, 56122 Pisa, Italy

Physiological systems are characterized by complex dynamics and nonlinear behaviors due to their intricate structural organization and regulatory mechanisms. Moreover, the optimization of physiological states and functions involves the continuous dynamic interaction of feedback mechanisms across different spatio-temporal scales. For this reason, advanced multivariate and multiscale approaches to biomedical signal analysis could strongly increase the information detectable from physiological signal monitoring, constituting a promising avenue to improve the knowledge of biological regulation in healthy and pathological states [1,2,3,4,5,6]. Moreover, thanks to the latest advances in technology that have provided miniaturized and high-performance acquisition systems, a synchronized multichannel recording of multiple signals—even in wearable and wireless mode—is currently possible. This Special Issue, therefore, focuses on original research papers dealing with computational methodologies for processing multivariate signals and the study of information across multiple time scales to characterize specific physiological states through linear and nonlinear interactions between components of the system. Research studies proposing novel multivariate or multiscale quantifiers and applying pattern-recognition algorithms to heterogeneous physiological data are presented in this sense.

In recent decades, multivariate and multiscale analyses have found fertile ground in the characterization of cardiovascular dynamics. It is clear, in fact, that processing more leads of the same systems or more signals from different organs and looking at the fractal properties of physiological dynamics can provide an undoubtedly more complete picture of the widespread phenomena of cardiovascular system complexity [1,7]. Such approaches will enhance the performance obtained through the analysis of cardiovascular signals in a completely non-invasive modality in characterizing aging and diagnosing cardiovascular diseases [8,9,10,11], and in the monitoring of emotions and mood disorders, which are strongly linked to changes in autonomic dynamics [12,13,14].

In the current Special Issue, multiscale partition-based Kolmogorov–Sinai (MKSE) entropy was presented [15]. The MKSE algorithm was used for the discrimination of elderly and young subjects in a resting state (by using the *Fantasia* dataset [16,17]) and the recognition of pathological dynamics due to congestive heart failure (CHF) and atrial fibrillation (AT) from a healthy condition (NS) (by using the *MIT-BIH* and *Congestive Heart Failure RR* datasets [17,18]). Statistical results showed that the MSKE method allowed for significantly discerning different conditions by cardiac dynamics, reporting a decrease in complexity in aging and cardiovascular diseases. These findings were consistent with previous studies; however, the proposed approach had the advantage of not requiring the tuning of embedding dimension value or other tolerances.

Frassineti et al. applied several multiscale entropy algorithms to heartbeat series of newborns to identify abnormal autonomic nervous system dynamics associated with seizures [19]. The multiscale approach applied to both sample entropy and fuzzy entropy algorithms [20,21] showed that it was possible to statistically discern seizure and seizure-free patients by using the Helsinki University public dataset [22]. The ictal events could not be detected by using the same entropy analyses at a single scale.

One of the most effective treatments for supraventricular arrhythmia is radiofrequency catheter ablation (RFA). This technique involves ablating abnormal electrical pathways in atrial tissues after analyzing intracardiac electrograms (EGMs). In [23], a new approach was proposed for a reliable estimation of electrical sources, accounting for the data uncertainty while analyzing EGMs. Starting from multivariate signals given by multiple catheter locations, the position of the electrical source was estimated through a multinomial distribution to model the activation probability of each sensor, recognizing the most probable path through a robust maximum-likelihood estimation.

In the early diagnosis of stenosis, a comprehensive study of acoustic pressure, flow fluctuations, and sound features related to stenosis progression was reported in [24]. The authors highlighted a relevant relationship between the severity level of stenosis progression and the frequency content of high-turbulence pressure fluctuations. Furthermore, their findings demonstrated that acoustic spatial-frequency maps could be used to assess the distance of the stenosis with respect to the measurement point.

Dresp-Langley et al. presented a multivariate analysis to investigate signals derived from multiple sensors located in a wireless wearable system to perform a non-invasive tracking of hand movements over time [25]. After a step-by-step statistical analysis, skill-specific differences were revealed in the functional organization of grip forces during the performance of complex precision tasks by individuals with varying expertise levels. Distinct co-variation patterns were identified in locally produced grip force signals acquired from sensors located in the middle, ring, and small fingers and the palm of the dominant hand. The results obtained were consistent with previous neuroimaging studies about grip force representations in the human brain.

Looking at the previous scientific literature, multivariate analysis of physiological signs is commonly linked to the application of pattern recognition algorithms in order to discern different conditions and predict the onset of pathological states or possible exacerbations.

In this Special Issue, two articles reported on machine learning models applied to multivariate datasets. The first study presented a machine learning approach for estimating mechanical ventilation parameters in treating various respiratory health concerns [26]. The proposed model was based on inverse mapping of artificial neural networks and used the Graded Particle Swarm Optimizer, a novel variant of the particle swarm optimization. Data from canine and feline patients at the University of Georgia College of Veterinary Medicine were used to train and test the machine learning approach. The findings showed that the proposed model could predict the mechanical ventilation parameters for several respiratory conditions.

Finally, Bizzego et al. used a multivariate dataset of six different physiological signals (electrocardiogram, electromyogram, electrodermal activity, photoplethysmogram, respiration, and acceleration) to test the performance of Deep Neural Networks (DNN) to recognize the signal type [27]. The signals in the dataset were collected from 232 subjects using four different acquisition devices. The signal types were optimally classified by the DNN (the performance was worse only for the respiratory signal, possibly due to the low number of samples), and the DNN features were used to train a support vector machine (SVM) model for the device recognition. Overall values of 0.638 and 0.609 were obtained for the multi-class Matthew Correlation Coefficient on the training and testing partitions, respectively.

In conclusion, the contributions to this Special Issue allowed a journey into various interesting scientific fields of application, where multivariate and multiscale approaches to data analysis can unveil features and dynamics inaccessible by using standard approaches based on single-channel or single-scale analyses. A common concept is evident from the studies: the multiplicity of information with different natures and observation scales can create a holistic vision of the problem to be described. These two approaches could be considered either as separate or as a whole. Each of these, taken separately, offers an enhancement of the level of the problem description and a deeper investigation of the phenomenon behind the measurements themselves. When they are considered together, the research space becomes more complex, requiring a structured strategy of analysis that has to be supported by a dedicated mathematical fashion and a clear vision of the implicit consequences that it will bring, not only in terms of the correctness of the formal results but also in the meanings assigned to the physiological processes that are the true sources of the information analyzed.
